# HOXA1 is overexpressed in oral squamous cell carcinomas and its expression is correlated with poor prognosis

**DOI:** 10.1186/1471-2407-12-146

**Published:** 2012-04-12

**Authors:** Carolina Cavalcante Bitu, Maria Fernanda de Souza Setúbal Destro, Manoela Carrera, Sabrina Daniela da Silva, Edgard Graner, Luiz Paulo Kowalski, Fernando Augusto Soares, Ricardo D Coletta

**Affiliations:** 1 Department of Oral Diagnosis, School of Dentistry, State University of Campinas, CP 52, CEP 13414-018 Piracicaba, São Paulo, Brazil; 2 Department of Head and Neck Surgery and Otorhinolaryngology, A. C. Camargo Hospital, CEP 01509-010 São Paulo, São Paulo, Brazil; 3 Department of Pathology, A. C. Camargo Hospital, CEP 01509-010 São Paulo, São Paulo, Brazil

**Keywords:** Oral cancer, HOXA1, Cellular proliferation, Prognosis

## Abstract

**Background:**

HOX genes encode homeodomain-containing transcription factors involved in the regulation of cellular proliferation and differentiation during embryogenesis. However, members of this family demonstrated oncogenic properties in some malignancies. The present study investigated whether genes of the HOXA cluster play a role in oral cancer.

**Methods:**

In order to identify differentially expressed HOXA genes, duplex RT-PCR in oral samples from healthy mucosa and squamous cell carcinoma was used. The effects of HOXA1 on proliferation, apoptosis, adhesion, invasion, epithelial-mesenchymal transition (EMT) and anchorage-independent growth were assessed in cells with up- and down-regulation of HOXA1. Immunohistochemical analysis using a tissue microarray (TMA) containing 127 oral squamous cell carcinomas (OSCC) was performed to determine the prognostic role of HOXA1 expression.

**Results:**

We showed that transcripts of HOXA genes are more abundant in OSCC than in healthy oral mucosa. In particular, HOXA1, which has been described as one of the HOX members that plays an important role in tumorigenesis, was significantly more expressed in OSCCs compared to healthy oral mucosas. Further analysis demonstrated that overexpression of HOXA1 in HaCAT human epithelial cells promotes proliferation, whereas downregulation of HOXA1 in human OSCC cells (SCC9 cells) decreases it. Enforced HOXA1 expression in HaCAT cells was not capable of modulating other events related to tumorigenesis, including apoptosis, adhesion, invasion, EMT and anchorage-independent growth. A high number of HOXA1-positive cells was significantly associated with T stage, N stage, tumor differentiation and proliferative potential of the tumors, and was predictive of poor survival. In multivariate analysis, HOXA1 was an independent prognostic factor for OSCC patients (HR: 2.68; 95% CI: 1.59-2.97; p = 0.026).

**Conclusion:**

Our findings indicate that HOXA1 may contribute to oral carcinogenesis by increasing tumor cell proliferation, and suggest that HOXA1 expression might be helpful as a prognostic marker for patients with OSCC.

## Background

Oral squamous cell carcinoma (OSCC), the most common cancer of the head and neck region, is the eighth most prevalent and accounts for 2% of all deaths by cancer worldwide [1]. OSCCs have a highly variable clinical course, and because it is often diagnosed only after it has reached an advanced stage, the overall survival rate is less than 50% in 5 years [2]. The most important prognostic factor for OSCC patients is still the clinical stage of disease (TNM stage), however, as survival for patients with the same disease stage varies considerably, better prognostic markers are needed.

The HOX gene family is composed of 39 genes organized into 4 chromosomal loci each containing anywhere from 9 to 11 genes [3,4]. These genes encode transcriptional factors that play important roles in organogenesis during development via regulation of proliferation, differentiation, survival, migration and invasion, among others [5]. Expression of HOX genes has been described in several adult tissues, where they perform important roles in maintaining homeostasis [6]. Interestingly, aberrant expression of numerous HOX genes has been observed in hematologic malignancies and various solid tumors, and recent studies have started to characterize the biological mechanisms related to their expression [7]. Little has been uncovered regarding the involvement of HOX genes in oral tumorigenesis [3]. In one previous study, we showed that most of the genes in the HOXB network are inactive in oral tissues, with the exception of HOXB2, HOXB7 and HOXB13, and that the misexpression of HOXB7 in OSCCs leads to increased tumor cell proliferation [8].

Few studies have suggested that HOXA1 plays a role in tumorigenesis. Besides being overexpressed in several tumors [5], HOXA1 influences numerous cellular processes including proliferation, apoptosis and epithelial-mesenchymal transition (EMT), and HOXA1 overexpression is sufficient for malignant transformation of nontumorigenic epithelial cells [9]. In the present study, we characterized the expression of the HOXA gene network in oral healthy mucosa and OSCC, and identified HOXA1 as a stimulator of cellular proliferation. We further showed that high immunoexpression of HOXA1 is significantly associated with shortened overall survival.

## Methods

### Samples and clinicopathological data

For the initial screening of the HOXA locus members, this study used fresh samples from healthy oral mucosa obtained from 10 patients without history of exposure to risk factors related to OSCC, such as smoking and alcohol consumption. Healthy mucosa specimens were obtained by 5 mm punch biopsy done 1 cm from the edge of irritation fibromas (hyperplastic lesions) of patients that were treated by conservative surgery. We have also used 14 pairs of fresh samples, each pair from the same patient, of OSCC and adjacent normal-looking oral mucosa. We have analyzed those samples by duplex reverse transcriptase-polymerase chain reaction (RT-PCR). Fresh samples were divided into two parts: one was fixed in formalin and embedded in paraffin for hematoxylin and eosin staining and immunohistochemistry, while the other was snapped frozen in liquid nitrogen.

To verify the clinicopathological correlation of HOXA1 expression, we used two tissue microarrays comprising 127 OSCC samples from patients diagnosed and treated at the Department of Head and Neck Surgery and Otorhinolaringology, A.C. Camargo Hospital, São Paulo, Brazil [10]. The OSCC patients (103 males and 24 females) showed a mean of 56.25 ± 10.53 years. History of alcohol consumption was recorded in 100 patients (78.74%) and tobacco smoking in 115 (90.55%) patients. The site of the primary tumor was predominantly the tongue (n = 91) and other sites such as the floor of mouth (n = 10), gingiva (n = 10), buccal mucosa (n = 9), the retromolar region (n = 6) and the lip (n = 2) accounted for the remaining cases. The patients were staged according to the International Union Against Cancer (TNM stage) as follows: T1 (n = 13), T2 (n = 33), T3 (n = 34) and T4 (n = 47), as well as N0 (n = 66) and N + (n = 61). All patients were staged as M0 at the time of diagnosis. Tumors were classified according to their pattern of cellular differentiation as previously described by Anneroth and collaborators [11]. After treatment, patients were followed up monthly and disease recurrence was histologically confirmed. Overall survival was calculated from date of diagnosis to death or last information, while disease-specific survival was determined from diagnostic date to death from cancer. Informed consent was obtained from each patient and the study was carried out with approval of the Human Research Ethics Committee.

### Cell culture and plasmids

The human OSCC cell line SCC9 was obtained from American Type Culture Collection (ATCC, Manassas, VA, USA), and cultured as recommended in a 1:1 mixture of Dulbecco's modified Eagle's media (DMEM) and Ham's F12 media (DMEM/F12; Invitrogen, Carlsbad, CA, USA) supplemented with 10% fetal bovine serum (FBS) and 400 ng/ml hydrocortisone (Sigma-Aldrich, St. Louis, MO, USA) at 37°C in a humidified atmosphere of 5% CO_2_. The HaCAT, a cutaneous normal epithelial cell line, was maintained in DMEM containing 10% FBS and antibiotics at 37°C in a 5% CO_2 _air atmosphere. HaCAT-HOXA1 stable cell lines were generated as previously described [12] using the HOXA1-pCMV Tag plasmid [13]. Control cells consisted of the HaCAT cells transfected with the vector alone (HaCAT-Control).

### Duplex RT-PCR

Total RNA from fresh tissues and cell lines was isolated with TRIzol reagent according to the manufacturer's protocol (Invitrogen). Following DNase I treatment, in order to eliminate genomic DNA contamination, 3 μg of total RNA per sample were used to generate cDNA using a superscript enzyme (Superscript II RT enzyme, Invitrogen). The resulting cDNAs were subsequently amplified, analyzed, and quantified as previously described [14]. Primer sequences, PCR conditions and the amplified lengths have been described [15], and glyceraldehyde-3-phosphate dehydrogenase (GAPDH) was used as a reference gene.

### Immunohistochemistry

HOXA1 and Ki67 immunostaining was performed using the streptavidin-biotin peroxidase complex method. Briefly, after dewaxing and hydration in graded alcohol solutions, the sections were treated with 3% H_2_O_2_, followed by antigen retrieval with 10 mM citric acid pH 6.0 in a pressure cooker. After washing with phosphate-buffered saline (PBS), the sections were treated with 1% bovine serum albumin (BSA) in PBS for 1 h, and then incubated with polyclonal rabbit anti-HOXA1 (Abcam, Cambridge, MA, USA), diluted 1:100, or monoclonal mouse anti-Ki67 (Dako Corp., Carpenteria, CA, USA) diluted 1:100, followed by the LSAB method (LSAB + System-HRP kit, Dako). Reactions were developed by incubating the sections with 0.6 mg/ml 3, 3'-diaminobenzidine tetrahydrochloride (Sigma-Aldrich) containing 0.01% H_2_O_2_. The control reactions were performed by the omission of the primary antibodies. The percentage of nuclear-positive cells was calculated with the aid of an image computer analyzer (Kontron 400, Carl Zeiss, Germany).

### Western blot analysis

Cells were washed with cold PBS and lysed in RIPA buffer (50 mM Tris-HCl pH 7.4, 150 mM NaCl, 1 mM EDTA, 1% NP-40, 1% deoxycholic acid, 0.5% sodium dodecyl sulfate, 1 mM phenymethylsulfony fluoride, 1 mM N-ethylmaleimide, 1 mM dithiothreitol, 10 μg/ml soybean trypsin inhibitor, 1 μg/ml leupeptin and 1 μg/ml aprotinin). After centrifugation, protein concentrations were measured using a protein assay according to the manufacturer's instructions (Bio-Rad Protein Assay, Bio-Rad, Hercules, CA). Eighty μg of total protein per sample were resolved in a 10% sodium dodecyl sulphate polyacrylamide gel electrophoresis (SDS-PAGE) under reducing conditions, and transferred to nitrocellulose membranes. The membranes were blocked for 2 h with 10% non-fat dry milk in PBS containing 0.1% Tween-20, rinsed in the same buffer, and incubated for 2 h with anti-FLAG M2 antibody (Stratagene) diluted 1:200 or with anti-β-actin antibodies (Sigma-Aldrich) diluted 1:70,000 in 5% milk in PBS. After washing, the membranes were developed using an Enhanced Chemiluminescent Western blot kit (GE Healthcare, Vienna, Austria). It was not possible to perform western blot analysis with the polyclonal rabbit anti-HOXA1 antibody.

### Bromodeoxyuridine-labeling (BrdU) index

Cells were plated in 8-well chamber slides at a density of 30,000 cells per well in 500 μl of medium containing 10% FBS. After 16 h, the cells were washed with PBS and cultured in serum-free medium for an additional 24 h. Following serum starvation, the medium was replaced by 10% FBS medium. Proliferation rates were determined 24 h after incubation by measuring BrdU incorporation into DNA. Briefly, BrdU antigen was added to the cultures and kept for 1 h at 37°C in 5% CO_2_. After incubation, cells were washed in PBS and fixed in 70% ethanol for 1 h. BrdU incorporation in proliferating cells was estimated using an immunohistochemical analysis kit (GE Healthcare). The BrdU-labeling index, expressed as the percentage of cells labeled with BrdU, was determined by counting 1,500 cells in 3 independent reactions using the Kontron 400 image analysis system (Zeiss).

### Ki67 index

Cells were cultured in culture chamber slides at 37°C in humidified air containing 5% CO_2 _for 24 h. After cellular synchronism and cell cycle induction as described above, the cells were fixed in 70% ethanol for 1 h and washed with PBS. Cells were then treated with 1% BSA diluted in PBS for 1 h, incubated with monoclonal antibodies against Ki67, and followed by the ABC method (StrepABC Complex/HRP, Dako). Reactions were developed with 0.6 mg/ml DAB containing 0.01% H_2_O_2_. The Ki67 index was calculated using an image analysis system by counting labeled nuclei of 1,500 cells in 3 independent reactions, and expressed as a percentage of Ki67-positive cells.

### Apoptosis analysis

The apoptosis index was determined by annexin V-FITC labeling [16]. Briefly, cells were harvested, washed with PBS and resuspended in the binding buffer (10 mM HEPES pH 7.4, 150 mM NaCl, 5 mM KCl, 1 mM MgCl_2 _and 1.8 mM CaCl_2_) containing annexin V-FITC at 1:500. After 20 min of incubation in the dark at room temperature, cells were also stained with propidium iodide (PI, Sigma-Aldrich). Apoptosis was analyzed on a FACScalibur flow cytometer equipped with an argon laser (Becton- Dickinson, San Jose, CA, USA) and quantified as the number of annexin V-FITC positive and PI negative cells divided by the total number of cells. A minimum of 10,000 events was analyzed in each sample.

### Cell adhesion assay

Wells of a 96-well culture plate were coated with 2 μg of type I collagen or fibronectin (BD Biosciences, Bredford, MA, USA) in 100 μl of PBS for 16 h at 4°C. The wells were washed 3 times with 200 μl of PBS and then coated with the same volume of 3% of BSA in PBS for 2 h at 37°C. Control wells were coated only with 3% BSA solution. HaCAT-HOXA1 and control cells were harvested and then resuspended in DMEM supplemented with 10% FBS and 3% BSA at a final concentration of 3 × 10^3 ^cells in 100 μl. The wells were washed and then 100 μl of the cell suspension was added to each well. The plate was then kept for 1 h in 37°C at 5% CO_2_. Loose cells were washed away and adhered cells were fixated in 10% formalin for 15 min. The cells were then stained with a solution of toluidin blue (1% toluidin blue and 1% borax). The excess dye was washed and the plate was incubated with a solution of 1% SDS for 5 min. Absorbance was measured at 650 nm. All the experiments were performed in quadruplicate.

### Analysis of EMT

The expression of EMT markers E-cadherin and β-cathenin in HaCAT-Control and HaCAT-HOXA1 clones was evaluated by western blot as described above. The antibodies anti-E-cadherin and anti-β-catenin were purchased from BD Biosciences and used at concentrations of 1:2500 and 1:1000, respectively.

### Invasion assay

Cell migration assays were done in modified Boyden chambers coated with Matrigel (BD Biosciences). Briefly, 6.5 nm Transwell chambers with 8 μm pores (Corning, NY, USA) were coated with Matrigel (BD Biosciences) diluted 1:1 in DMEM for 2 h at 37°C. Serum starved cells (8 × 10^4 ^cells/well) were plated to the upper chamber in 200 μl of serum-free DMEM. The lower chamber was filled with 500 μl of DMEM supplemented with 10% FBS. After incubation of 72 h, nonmigratory cells in the upper chamber were gently removed with a cotton swab and cells that migrated to the bottom of the membrane were fixed and stained with a solution of 1% toluidin blue. Absorbance was measured at 650 nm.

### Soft agar assays

HaCAT-HOXA1 and HaCAT-Control clones were plated in DMEM with 10% FBS and 0.4% agar. SCC-9 cell line was plated in DMEM/F12 with 10% FBS, 400 ng/ml hydrocortisone and 0.4% agar. Both lines were incubated at 37°C in 5% CO_2_. The cells in agar were overlaid with fresh growth medium every 2 to 3 days and colony formation was assessed after 4 weeks.

### Small interference RNA (siRNA) mediating HOXA1 silence

To determine the role of endogenous HOXA1, we examined the effect of HOXA1 siRNA transfection on SCC9 proliferation. Three sequences targeting HOXA1 were chemically synthesized, annealed and purified by the manufacturer (Invitrogen). In essence, cells grown to 50% confluence were transfected with 100 nM of a mixture containing equal parts of the 3 HOXA1 siRNAs using a liposome method according to the manufacturer's instructions (Lipofectamine 2000, Invitrogen). In parallel, to act as negative controls, cells were transfected with a non-specific siRNA or with the transfectant reagent only (mock transfection). Forty-eight hours after transfection, the efficacy of the HOXA1 knock down was determined by duplex-RT-PCR. Proliferation assays were performed as described above.

### Statistical analysis

*In vitro *assays were analyzed using the Kruskal-Wallis test. Correlations between immunohistochemical expression of HOXA1 and clinicopathological parameters of the tumors were performed by cross-tabulation and standard chi-square test. For these analyses, the tumors were divided into 2 groups, with low and high HOXA1 expression, based on the median value of HOXA1-positive cells. Survival curves were constructed based on the Kaplan-Meier method and compared with the Log-rank test. For univariate and multivariate survival analysis, the Cox proportional hazard model was employed including variants that showed a significant correlation with HOXA1. The level of significance considered was 5% (p ≤ 0.05).

## Results

### Differential expression of HOXA1 between healthy oral mucosa and OSCC

**Figure 1:**
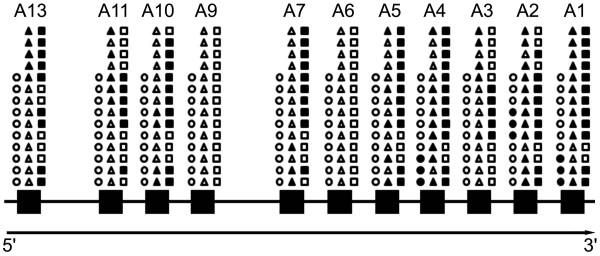
**Diagram of expression of the members of HOXA locus in samples from healthy oral mucosa, normal-looking oral mucosa adjacent to OSCC, and OSCC**. Each symbol represents one specific sample, and their positions are preserved throughout the image. Circles represent healthy oral mucosa derived from patients without contact with the main oral cancer risk factors, and triangles and squares represent normal-looking mucosa and OSCC from the same patient, respectively. Open symbols indicate silent (inactive) HOXA genes, whereas closed symbols indicate active genes. Note the abundance of active genes in OSCC samples.

**Figure 2:**
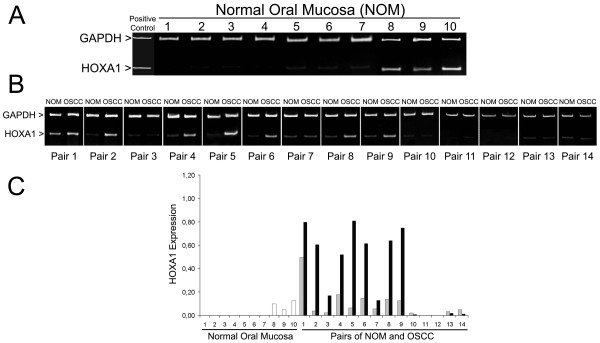
**HOXA1 is overexpressed in OSCCs**. **(A) **Analysis of HOXA1 and GAPDH by duplex RT-PCR on samples of healthy oral mucosa from patients without contact with the main risk factors for oral cancer and **(B) **on pairs of OSCC and adjacent normal-looking oral mucosa from the same patient. **(C) **Densitometric analysis of the HOXA1 bands demonstrated a significant higher expression in OSCCs compared to healthy oral mucosas from patients without recognized OSCC-risk factors (p < 0.001).

In order to determine the expression of the genes in the HOXA locus, total RNA was extracted from tissue samples and RT-PCR was performed using specific oligonucleotides designed against each of the genes in the HOXA locus and GAPDH. Figure 1 depicts the diagram of HOXA gene expression in the healthy oral mucosa, normal-looking oral mucosa near OSCC and OSCC samples. Two out of 11 genes (HOXA6 and HOXA9) were silenced in all samples of this study. The majority of the healthy oral mucosa samples from patients not exposed to recognized OSCC risk factors did not express HOXA genes, with the exception of HOXA1, HOXA2 and HOXA4, which were expressed by 30% of the samples. The expression in normal-looking oral mucosa near OSCC samples was more abundant when compared to the expression observed in OSCC samples. However, considering the intensity levels of gene expression, as represented by the densitometric ratio of the optical density of target transcript/GAPDH bands, we identified that the expression of HOXA4, HOXA5, HOXA7 and HOXA10 was significantly higher in OSCC samples compared to both healthy oral mucosa and normal-looking mucosa near OSCC. The expressions of HOXA1, HOXA2 and HOXA13 were significantly higher in OSCC samples when compared to healthy oral mucosa. Of particular interest, the expression of HOXA1 was also found to be significantly higher in normal-looking oral mucosa near OSCC than in healthy oral mucosa (Figure 2). Since HOXA1 expression was associated with tumors from different organs, and its dysregulated expression can influence numerous cellular processes related to tumorigenesis and was correlated with poor prognosis, we further analyzed the role of HOXA1 in OSCC.

**Figure 3:**
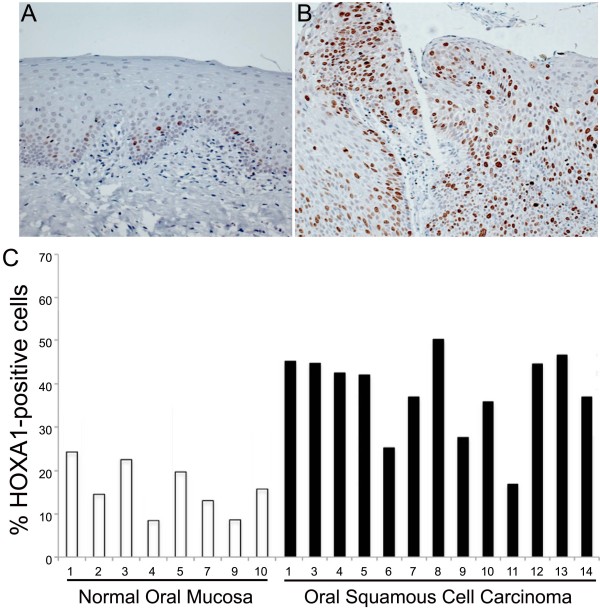
**Immunohistochemical detection of HOXA1 in healthy oral mucosa and OSCC**. Representative samples of healthy oral mucosa **(A) **and OSCC **(B) **of this study. In the healthy mucosa, HOXA1 was clearly limited to the nucleus of the epithelial cells located in the basal and suprabasal layers, whereas in the OSCCs, HOXA1-positive cells were broadly observed in the tumor (Original magnification x200). **(C) **The percentage of HOXA1-positive cells was significantly higher in OSCCs than in healthy oral mucosas (p < 0.001).

To confirm the higher expression of HOXA1 in OSCC samples compared to healthy oral mucosa, we performed immunohistochemical analysis. Immunoreactivity for HOXA1 was observed as a nuclear stain restricted to the basal and suprabasal layers in healthy mucosas, whereas a broad positivity with variable distribution and intensity was found in the OSCC samples (Figure 3). Our results also showed that OSCC samples have a higher mean percentage of HOXA1-positive cells than healthy mucosa samples (p < 0.01).

### HOXA1 promotes cellular proliferation

**Figure 4:**
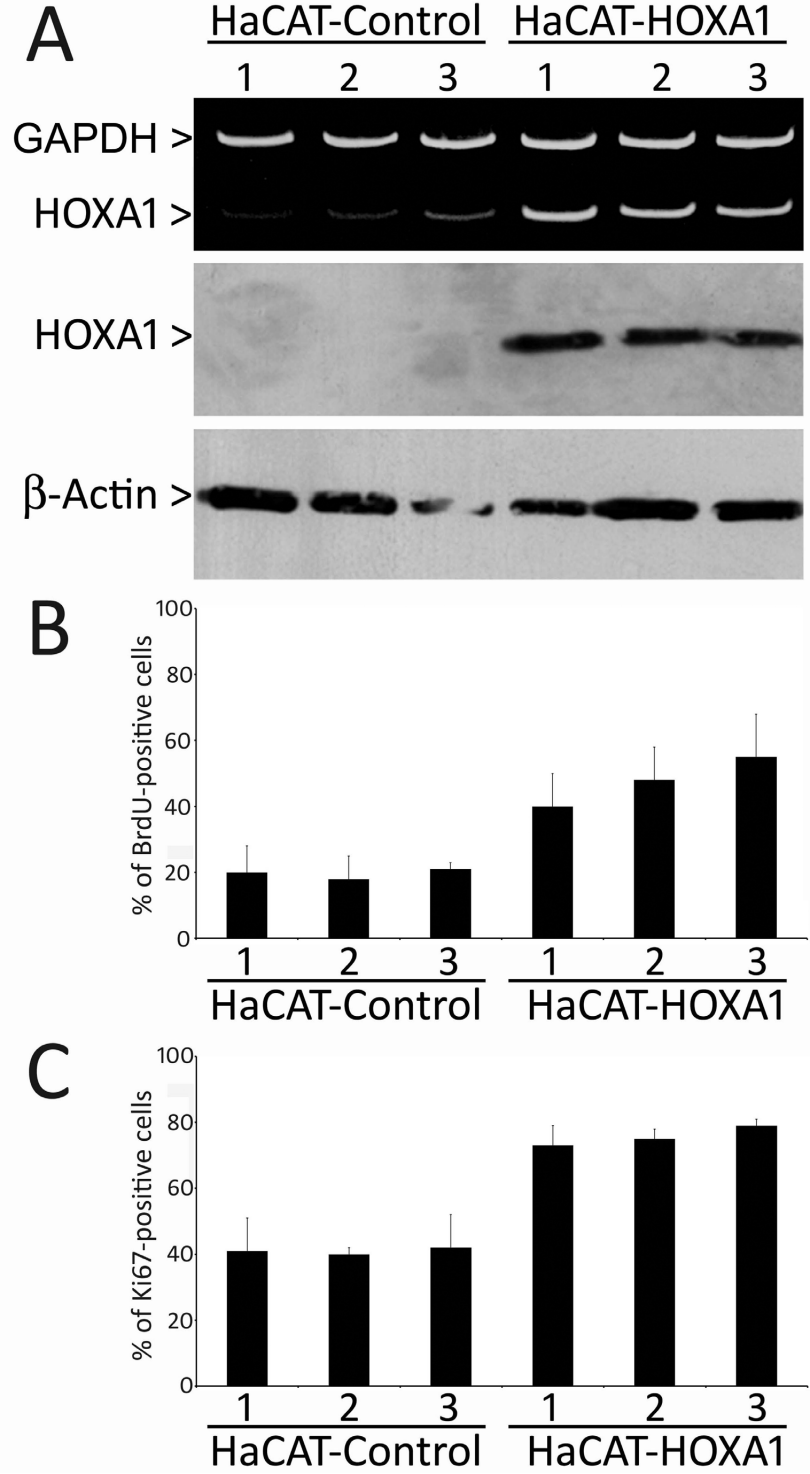
**Overexpression of HOXA1 induces cellular proliferation**. **(A) **Representative duplex RT-PCR and western blot analysis of HOXA1 in HaCAT-Control and HaCAT-HOXA1 transfectants, revealing an increase in HOXA1 levels in overexpressing clones. Assays measuring BrdU incorporation **(B) **and Ki67 expression **(C) **demonstrated that HOXA1 overexpressing cell lines have a statistically significant increase in proliferation as compared to control cells (for BrdU index p < 0.01 between groups, and for Ki67 index p < 0.05 between groups). The labeling index of BrdU and Ki67 corresponds to the mean percentage of positive cells of 3 experiments for each cell line.

**Figure 5:**
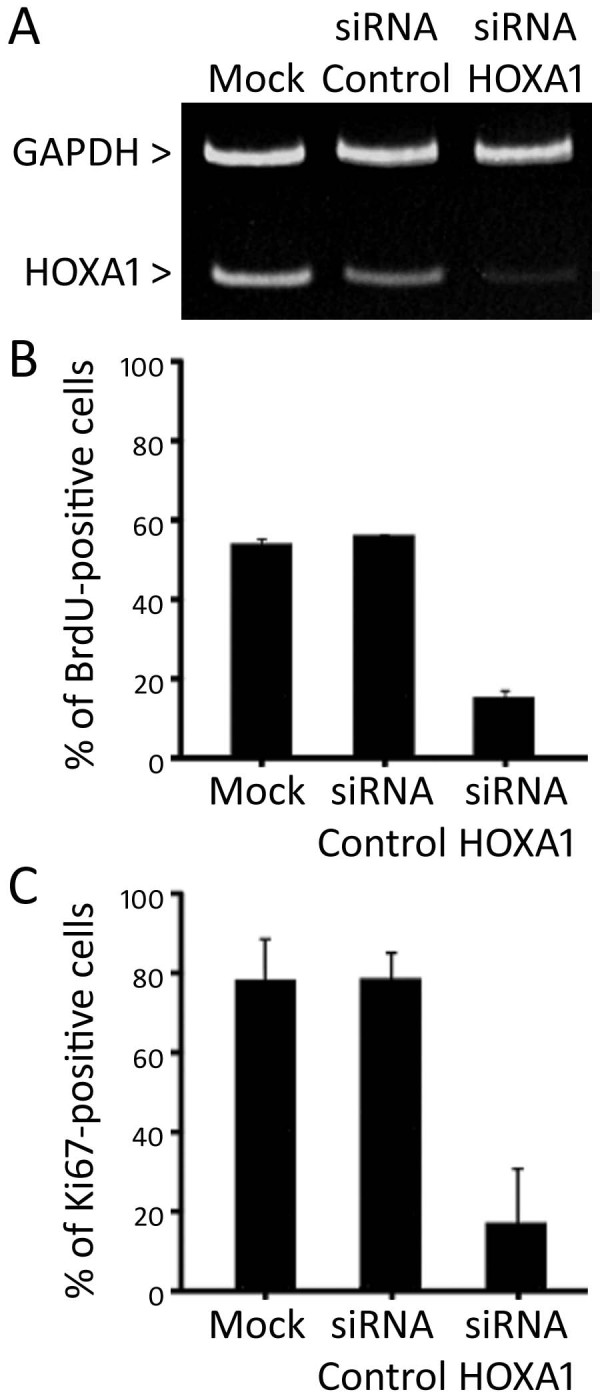
**Inhibition of HOXA1 by siRNA in SCC9 cells decreases cellular proliferation**. **(A) **siRNA working against HOXA1 significantly decreased HOXA1 levels as revealed by representative duplex RT-PCR. **(B) **BrdU incorporation and Ki67 immunocytochemical expression **(C) **assays showed a statistically significant decrease in proliferation when HOXA1 was downregulated via siRNA (for BrdU index p < 0.01 and for Ki67 index p < 0.05). Data correspond to the mean percentage of positive cells of 3 experiments.

To better understand the role of HOXA1 in the events that control oral tumorigenesis, we overexpressed HOXA1 in the HaCAT epithelial cell line, which shows very low levels of its expression. Stable HOXA1 and control transfectants were generated and examined for HOXA1 mRNA and protein levels. Three stable HOXA1-overexpressing clones (HaCAT-HOXA1) and 3 control clones (HaCAT-Control) were chosen for further analyses (Figure 4A). HOXA1-overexpressing cells showed a statistically significant increase in proliferation when compared to HaCAT-Control cells, as assessed by both BrdU incorporation (p < 0.01) and Ki67 expression (p < 0.05) indexes (Figure 4B and 4C). In order to confirm these findings, we next knocked down HOXA1 levels in SCC9 cells, which have high endogenous levels of HOXA1. Either specific-stranded RNA oligonucleotides against HOXA1 or negative RNA control were transfected into SCC9 cells. When HOXA1-specific oligonucleotides were used, a rapid downregulation of HOXA1 mRNA was detected (Figure 5A). The decrease in HOXA1 levels resulted in a concomitant decrease in proliferation. Both cell proliferation assays (BrdU incorporation and Ki67 expression) showed a statistically significant decrease in proliferation when HOXA1 was downregulated with siRNA (p < 0.01 for BrdU index and p < 0.05 for Ki67 index; Figure 5B and 5C).

To further characterize the tumorigenic effects of HOXA1 overexpression, we carried out *in vitro *assays to measure apoptosis, adhesion, invasion, expression of EMT markers, and independent-anchorage growth in soft-agar. Unexpectedly, HOXA1 overexpression did not significantly modulate those events when compared with control clones (Additional file 1: Figure S1, Additional file 2: Figure S2, Additional file 3: Figure S3, Additional file 4: Figure S4, Additional file 5: Figure S5).

### HOXA1 expression is associated with clinicopathological features of OSCCs

To investigate whether HOXA1 expression is associated with clinicopathological features of OSCC patients, we performed immunohistochemistry on 127 human OSCCs to examine HOXA1 expression concurrent with the proliferative marker Ki67. Parallel tissue microarray slides were analyzed for HOXA1 and Ki67 expression. The clinicopathological correlations with the expression of HOXA1 are described in Table 1. A high percentage of HOXA1-positive cells was significantly associated with T stage (p = 0.039), N stage (p = 0.046) and tumor cellular differentiation (p = 0.026). The expression of HOXA1 was not correlated with age, gender, ethnicity, tumor localization, alcohol or tobacco consumption, location of the tumor, and vascular or neural infiltration. The proliferation rate of the tumors was evaluated through the labeling index for Ki67. The median (23%) was used to divide the samples into two groups: high and low expression on Ki67, which were then associated with HOXA1 immunopositivity. OSCC samples that overexpressed HOXA1 showed statistically significant increase in the percentage of Ki67-positive cells compared with low expressing tumors (p = 0.0001) (Table 1).

**Table 1:** Correlation between HOXA1 immunoexpression and clinicopathological variables of 127 OSCCs

Parameter	% of positive cells		p value
	< 37	≥ 37	
	n (%)	n (%)	
Age			
< 56 years	37 (48.68)	26 (50.98)	0.79
≥ 56 years	39 (51.32)	25 (49.02)	

Gender			
Male	60 (78.94)	43 (84.31)	0.44
Female	16 (21.06)	8 (15.69)	

Ethnicity			
Caucasian	64 (84.21)	44 (86.27)	0.75
Non-Caucasian	12 (15.79)	7 (13.73)	

Smoking habit			
No	8 (10.52)	4 (7.84)	0.61
Yes	68 (89.48)	47 (92.16)	

Drinking habit			
No	14 (18.42)	13 (25.49)	0.34
Yes	62 (81.58)	38 (74.51)	

Location			
Tongue	53 (69.73)	38 (74.5)	0.55
Other	23 (30.27)	13 (25.5)	

T Stage			
T1 + T2	33 (43.42)	13 (25.49)	0.039
T3 + T4	43 (56.58)	38 (74.51)	

N Stage			
N0	45 (59.21)	21 (41.17)	0.046
N+	31 (40.79)	30 (58.83)	

Histopathological grade			
WD + MD	48 (63.15)	22 (43.13)	0.026
Undifferentiated	28 (36.85)	29 (56.87)	

Vascular infiltration			
No	48 (63.16)	33 (64.7)	0.85
Yes	28 (36.84)	18 (35.3)	

Neural Infiltration			
No	44 (57.89)	36 (70.58)	0.14
Yes	32 (42.11)	15 (29.42)	

Ki-67 positive cells			
< 23%	56 (73.68)	14 (27.45)	0.0001
≥ 23%	20 (26.32)	37 (72.55)	

### High immunoexpression of HOXA1 is associated with shortened overall survival

**Figure 6:**
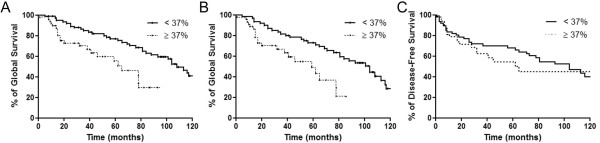
**Expression of HOXA1 is associated with shortened overall survival of patients with OSCC**. **(A) **The overall survival analysis according to the Kaplan-Meier method for HOXA1 immunoexpression revealed that high HOXA1 expression is associated with poor prognosis with a 5-year survival of 55.5% (p = 0.0073). **(B) **Disease-specific overall survival for patients with high expression of HOXA1 was even shorter (p = 0.0048). **(C) **Five-year disease-free survival was not associated with HOXA1 immunoexpression in OSCC tumors (p = 0.27).

High HOXA1 immunoreactivity was a marker of reduced overall survival with a 5-year survival of 55.5% (95% CI 42.4-76.4) for the patients with strong positivity for HOXA1 compared with 76.8% (95% CI 60.7-88.1) for those with low HOXA1 expression (p = 0.007; Figure 6A). The 5-year disease-specific survival was even shortened. Patients with high HOXA1 expression showed a 5-year disease-specific survival of 49.2% (95% CI 32.3-61.7) compared with 72.8% (95% CI 62.3-91.3) for patients with low HOXA1 immunoreactivity (p = 0.0048; Figure 6B). No significant influence of HOXA1 immunoexpression in the disease-free survival was observed in this cohort (Figure 6C). Multivariate analysis was performed to assess the independent predictive value of HOXA1, including variables that demonstrated a statistical correlation with HOXA1. In this analysis, high HOXA1 immunopositivity with a hazard ratio (HR) of 2.68 (95% IC 1.59-2.97, p = 0,026) and regional metastasis at diagnosis (N stage) with a HR of 1.74 (95% IC 1.20-2.52, p = 0.017) remained as independent prognostic factors (Table 2).

**Table 2:** Cox regression analysis for overall survival of OSCC patients

Parameter	Overall survival in 5 years (%)	HR (95% CI)/p value	
		Univariate	Multivariate
HOXA1			
< 37%	76.88	Reference	Reference
≥ 37%	55.55	3.74 (1.66-4.50)/0.007	2.18 (1.59-2.97)/0.026

T Stage			
T1/T2	78.26	Reference	Reference
T3/T4	63.91	1.36 (0.98-1.90)/0.28	1.22 (0.89-1.68)/0.46

N Stage			
N0	77.71	Reference	Reference
N+	64.77	2.79 (1.38-2.78)/0.004	1.74 (1.20-2.52)/0.017

Histopathological grade			
WD + PD	70.67	Reference	Reference
Undifferentiated	68.49	1.12 (0.89-1.68)/0.55	1.36 (0.98-2.06)/0.86

Ki-67 positive cells			
< 23%	67.83	Reference	Reference
≥ 23%	66.83	1.21 (0.78-1.75)/0.46	1.07 (0.78-1.35)/0.70

## Discussion

Aberrant expression of homeobox genes is common in cancers [5], and recent studies have started to elucidate the causal role of these genes in tumorigenesis. In general, homeobox genes regulate developmental programs that coordinate different cell behaviors during embryogenesis, but their misexpression in differentiated cells can result in the acquisition of tumor-promoting properties, including proliferation, dedifferentiation, migration, invasion and survival [17]. Good examples of the causal effects of homeobox genes were obtained in animal models with Oct-4, which promoted testicular germ cell tumors when expressed inappropriately [18]; NKX3.1, which induced prostatic intraepithelial neoplasia and enhanced prostate cancer progression in collaboration with loss of function of the PTEN tumor-suppressor gene [19]; and SIX1, the overexpression of which induced breast cancer in mammary epithelial cells [20].

In the present study we identified differentially expressed genes belonging to cluster A of the HOX family of homeobox genes in OSCC compared to healthy oral mucosa. In healthy oral mucosa derived from patients without risk factors for oral cancer, only HOXA1, HOXA2 and HOXA4 were expressed, whereas the abundance of transcripts in histologically normal-looking oral mucosa near OSCC and in OSCC samples was higher. Among the transcripts detected in this study, the expression of HOXA1 was significantly higher in OSCC compared with healthy oral mucosa. In addition to the high transcriptional levels, we showed that HOXA1 homeoprotein expression was limited to the basal and suprabasal cells of the normal epithelium, while OSCCs showed broad immunoreactivity in the tumor nests and a significantly higher number of HOXA1-positive cells than that of controls. HOXA1 misexpression has been reported in leukemia, and carcinomas of the cervix, breast and oral cavity [21]. In the latter, similar to our findings, it was demonstrated that the expression of HOXA1 was significantly higher in OSCCs when compared with healthy oral mucosas [3]. The present study further revealed that the expression of HOXA1 was correlated with Ki67 immunohistochemical expression in both control and OSCC tissues and that the forced expression of HOXA1 in the non-tumorigenic cell line HaCAT was able to stimulate cell cycle progression, whereas the downregulation of HOXA1 levels in the OSCC cell line SCC9 decreased the proliferation.

Evidence from both *in vitro *and *in vivo *studies has suggested an oncogenic role for HOXA1 based on its effects on the promotion of anchorage-independent growth of normal epithelial cells and on induction of tumors in mice [22]. In hematopoietic cells, overexpression of HOXA1 blocked differentiation, leading to transformation by colony formation in soft agar assays and to the development of acute myeloblastic leukemia in lethally irradiated mice [21]. In immortalized mammary epithelial cells, expression of HOXA1 resulted in a dramatic increase in anchorage-independent proliferation by the promotion of cell survival mediated by activation of the STAT pathway [13] and by transcriptional upregulation of Bcl-2 [22]. Furthermore, HOXA1 is a downstream effector of E-cadherin-directed signaling required for anchorage-independent proliferation of mammary carcinoma cells [9]. In addition, in growth hormone-induced oncogenic transformation of immortalized human mammary epithelial cells, HOXA1 governs the transcriptional up-regulation of c-Myc, cyclin D1 and Bcl-2 that are required for this event [23]. In contrast to those studies, overexpression of HOXA1 did not affect apoptosis, adhesion, invasion, EMT and anchorage-independent growth of HaCAT epithelial cells. Reasons for the lack of those phenotypes in HOXA1 overexpression cells could reside in the fact that many of the members of homeobox gene families required cofactors for their complete functional activity [24]. HOXA1 is dependent on the MEIS, PREP or HTH protein cofactors to activate and/or repress transcription [25]. Consistent with this observation, Meis1 augmented the efficacy of HOXA1 on tumorigenic induction of primary hematopoietic cells, resulting in a more intense growth and increased colony number in soft agar assays [21]. Thus, it is possible that the availability of cofactors in the HaCAT cells, as well as their stoichiometry with HOXA1, was not conducive to stimulating those biological functions of the protein, thereby removing the ability of aberrant HOXA1 expression to induce anchorage-independent growth and inhibit apoptosis.

Next, we investigated the clinicopathological relevance of HOXA1 expression for OSCC patients. Although 100% of the samples demonstrated positivity for HOXA1, the levels of immunoexpression varied considerably. We have observed that higher levels of HOXA1 were associated with larger tumor size at diagnosis, histopathologic differentiation of the tumor with high positivity for HOXA1 in undifferentiated tumors, and higher proliferative potential of the tumor. These tumor progression hallmarks were consistent with what was already described as induced by HOXA1 overexpression in other cell lines [9,13,21,22]. Our findings also show a significant association between high HOXA1 expression with the presence of lymph node metastasis and worse clinical outcome, where patients with a high number of HOXA1-positive cells had substantially shorter overall and cancer-specific survival than did patients with a low number of HOXA1-positive cells. Furthermore, multivariate analysis showed that HOXA1 overexpression was an independent marker for overall survival in the entire sample after adjusting for other prognostic factors. Although a strong association in vitro between HOXA1 and Ki-67 was observed, Ki-67 immunohistochemical expression was not correlated with the overall survival of patients with OSCC. Interestingly, this lack of association has been reported by other studies [26,27]. We do not feel that HOXA1 was sufficiently studied in its role in tumor prognosis in OSCC or other malignancies, however, several studies have reported that lymph node metastasis is the most reliable marker of OSCC patient prognosis [28-30], and our findings showed that the presence of lymph node metastasis significantly correlated with high HOXA1 expression and both were significant prognostic factors for overall survival by multivariate analysis. Since HOXA1 is expressed in a number of cancers, our findings encourage its verification and monitoring in other malignancies.

In addition, as master regulators of development and tissue homeostasis, HOX genes often rely on other homeobox genes, as well as closely related HOX members, to ensure tissue specificity via gene expression regulation [5]. Therefore, it is common to have dysregulated expression of several HOX genes at the same time during the malignancy process [3,21,31]. Our group has previously demonstrated the role of HOXB7 and its prognostic significance to OSCC [8,32]. In the latter study, immunoexpression of HOXB7 was significantly associated with clinically important markers of OSCC behavior, including lymph nodal metastasis at diagnosis, vascular infiltration and proliferative potential of the tumor, resulting in significantly shortened overall survival. Interestingly, 110 samples used in the present study were also utilized to analyze HOXB7, and a strong and positive correlation between these two markers was observed in the OSCC samples (data not shown), highlighting the importance of several members of the HOX family to OSCC.

## Conclusion

In closing, to our knowledge, our data are the first to demonstrate that HOXA1 expression correlates with OSCC patient outcome and provides an opportunity of considering its potential clinical applications as a prognostic marker. Although there have been considerable advances in treatment protocols for OSCC patients, their overall survival in five years still remains between 50-60% [33]. Therefore, a greater understanding and knowledge of the biological events that precede the clinical presentation of the disease can contribute to more individualized treatment of patients affected by OSCC.

## Competing interests

The authors declare that they have no competing interests.

## Authors' contributions

CCB, MFSSD and RDC designed the study and drafted the paper. SDS, EG, LPK and FAS collected the samples and provided the clinical and histopathological data. CCB, MFSSD, MC performed the experiments. RDC and SDS conducted statistical analysis. All authors revised the final version of the manuscript. All authors read and approved the final manuscript.

## Pre-publication history

The pre-publication history for this paper can be accessed here:

http://www.biomedcentral.com/1471-2407/12/146/prepub

## Supplementary Material

Additional file 1 **Figure S1 Apoptosis levels in HaCAT-Control and HaCAT-HOXA1 clones**. Cells were cultured for 24 h and then stained with annexin V and propidium iodide to estimate dead cells. The number of apoptotic cells was very low in both control and HOXA1 overexpressing clones. A, B and C are representative histograms of HaCAT-Control clones, and D, E and F are representative HaCAT-HOXA1 cells. The assay was performed three times. Click here for file

Additional file 2 **Figure S2 Effect of HOXA1 overexpression on adhesion of HaCAT cells to extracellular matrix substrates**. HaCAT-Control and HaCAT-HOXA1 cells were harvested and allowed to adhere for 1 h to wells of a 96-well plate coated with type I collagen or fibronectin. Untreated surface was used as a control. Non-adherent cells were washed away, and the number of adherent cells was determined by toluidin blue stain. As expected, both extracellular matrix proteins increased the adhesion, but no differences between HOXA1 overexpressing clones and controls were observed. Click here for file

Additional file 3 **Figure S3 Effect of HOXA1 overexpression on markers of epithelial-mesenchymal transition**. Western blot analysis for E-cadherin and β-catenin revealed that overexpression of HOXA1 was not capable of inducing epithelial-mesenchymal transition. Click here for file

Additional file 4 **Figure S4 Overexpression of HOXA1 does not modulate invasion of HaCAT cells**. HaCAT-Control and HaCAT-HOXA1 cells were seeded into the upper chamber of transwell inserts; media with 10% FBS was used as a chemotactic agent in the lower chamber; and the cells were cultured for 72 h. Invading cells were estimated by toluidin blue stain. SCC9 cells were used as the positive control. Click here for file

Additional file 5 **Figure S5 Overexpression of HOXA1 does not confer the ability to form colonies in soft agar**. HaCAT-Control and HaCAT-HOXA1 cells were plated in triplicate wells in 0.4% agar and allowed to grow for 4 weeks. SCC9 cells were used as the positive control. This experiment was reproduced 3 times. Panels A-C represent SCC9 cells, D-F represent HaCAT-Control clones, and G-I correspond to HaCAT-HOXA1 clones. Click here for file
