# Supplementary figures and images for: HOXA1 is overexpressed in oral squamous cell carcinomas and its expression is correlated with poor prognosis
Source: BMC Cancer. 2012 Apr 12;12:146. doi: 10.1186/1471-2407-12-146 (PMC3351375; doi:10.1186/1471-2407-12-146)

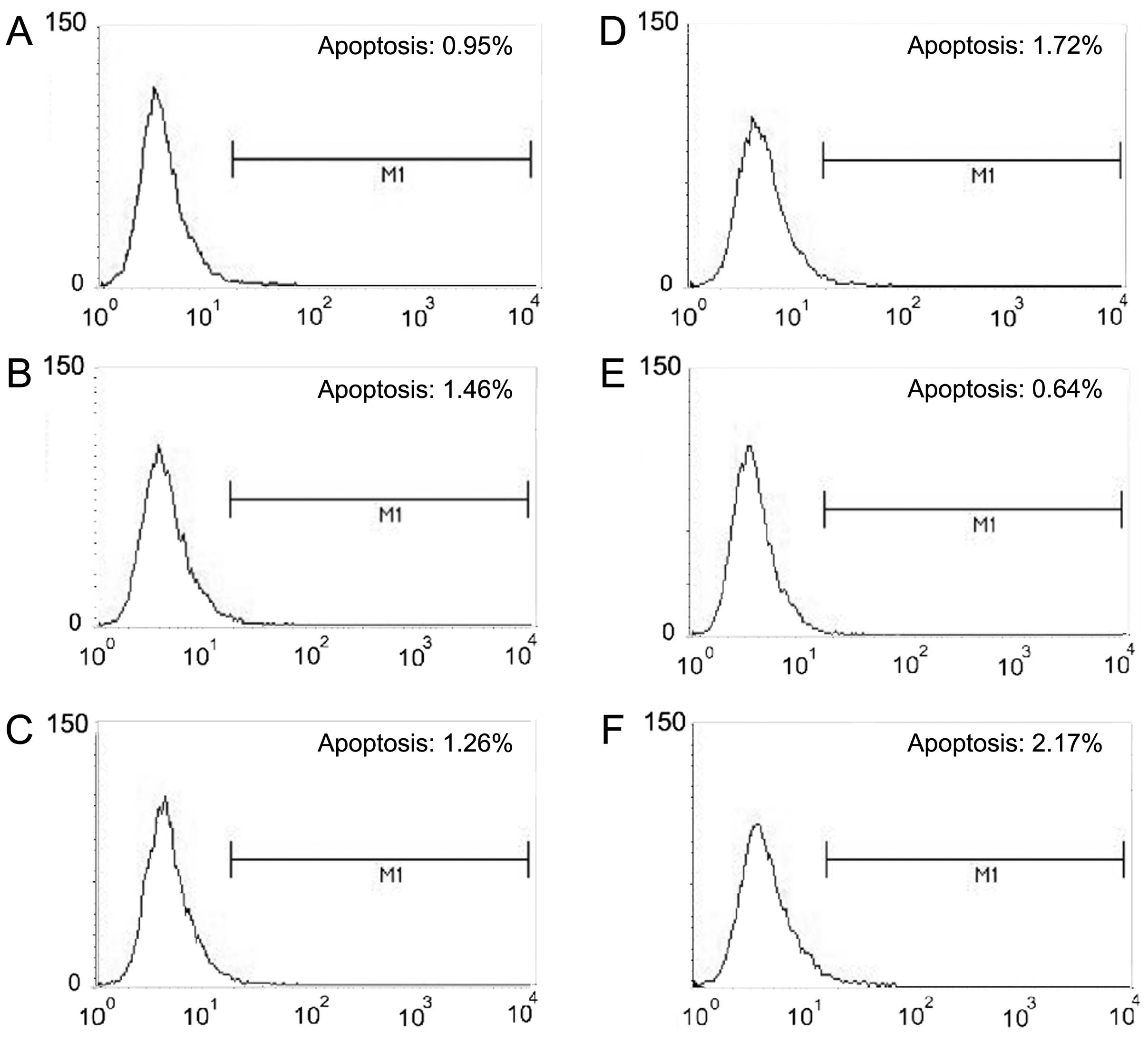

Supplement: Additional file 1 — Figure S1 Apoptosis levels in HaCAT-Control and HaCAT-HOXA1 clones. Cells were cultured for 24 h and then stained with annexin V and propidium iodide to estimate dead cells. The number of apoptotic cells was very low in both control and HOXA1 overexpressing clones. A, B and C are representative histograms of HaCAT-Control clones, and D, E and F are representative HaCAT-HOXA1 cells. The assay was performed three times. [file 1471-2407-12-146-S1.JPEG]

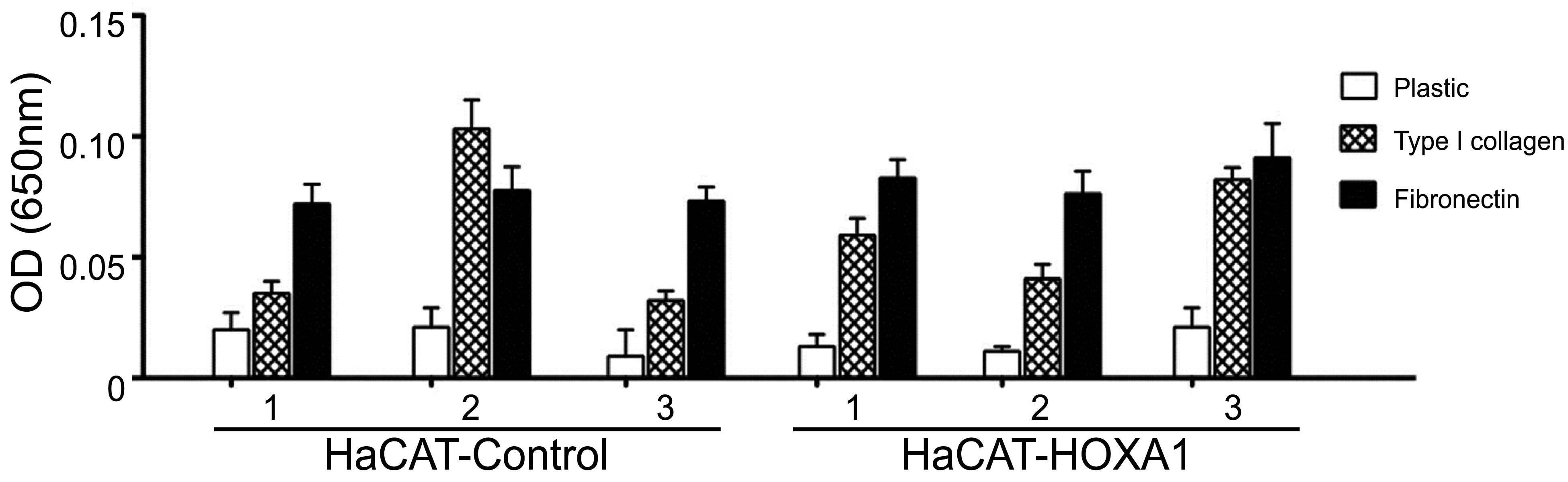

Supplement: Additional file 2 — Figure S2 Effect of HOXA1 overexpression on adhesion of HaCAT cells to extracellular matrix substrates. HaCAT-Control and HaCAT-HOXA1 cells were harvested and allowed to adhere for 1 h to wells of a 96-well plate coated with type I collagen or fibronectin. Untreated surface was used as a control. Non-adherent cells were washed away, and the number of adherent cells was determined by toluidin blue stain. As expected, both extracellular matrix proteins increased the adhesion, but no differences between HOXA1 overexpressing clones and controls were observed. [file 1471-2407-12-146-S2.JPEG]

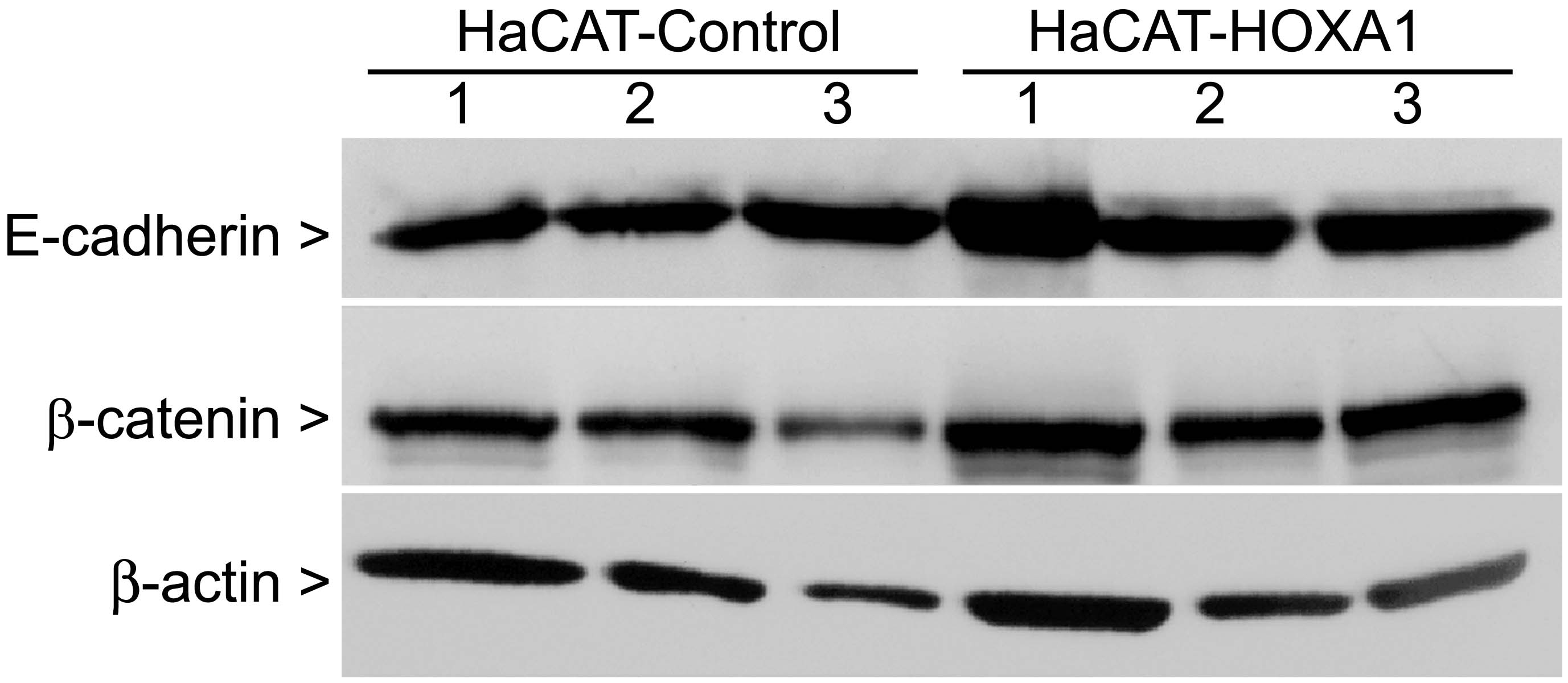

Supplement: Additional file 3 — Figure S3 Effect of HOXA1 overexpression on markers of epithelial-mesenchymal transition. Western blot analysis for E-cadherin and β-catenin revealed that overexpression of HOXA1 was not capable of inducing epithelial-mesenchymal transition. [file 1471-2407-12-146-S3.JPEG]

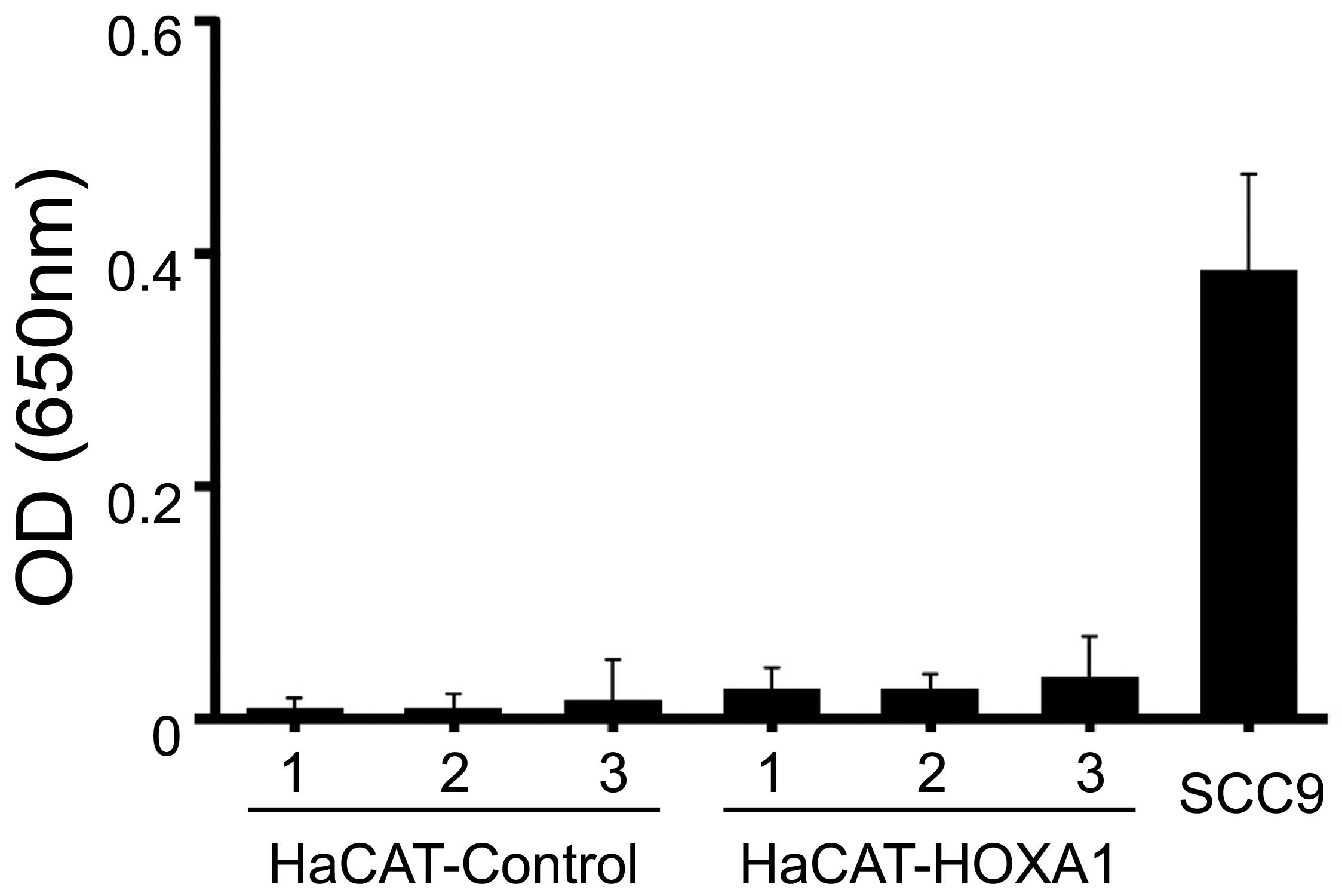

Supplement: Additional file 4 — Figure S4 Overexpression of HOXA1 does not modulate invasion of HaCAT cells. HaCAT-Control and HaCAT-HOXA1 cells were seeded into the upper chamber of transwell inserts; media with 10% FBS was used as a chemotactic agent in the lower chamber; and the cells were cultured for 72 h. Invading cells were estimated by toluidin blue stain. SCC9 cells were used as the positive control. [file 1471-2407-12-146-S4.JPEG]

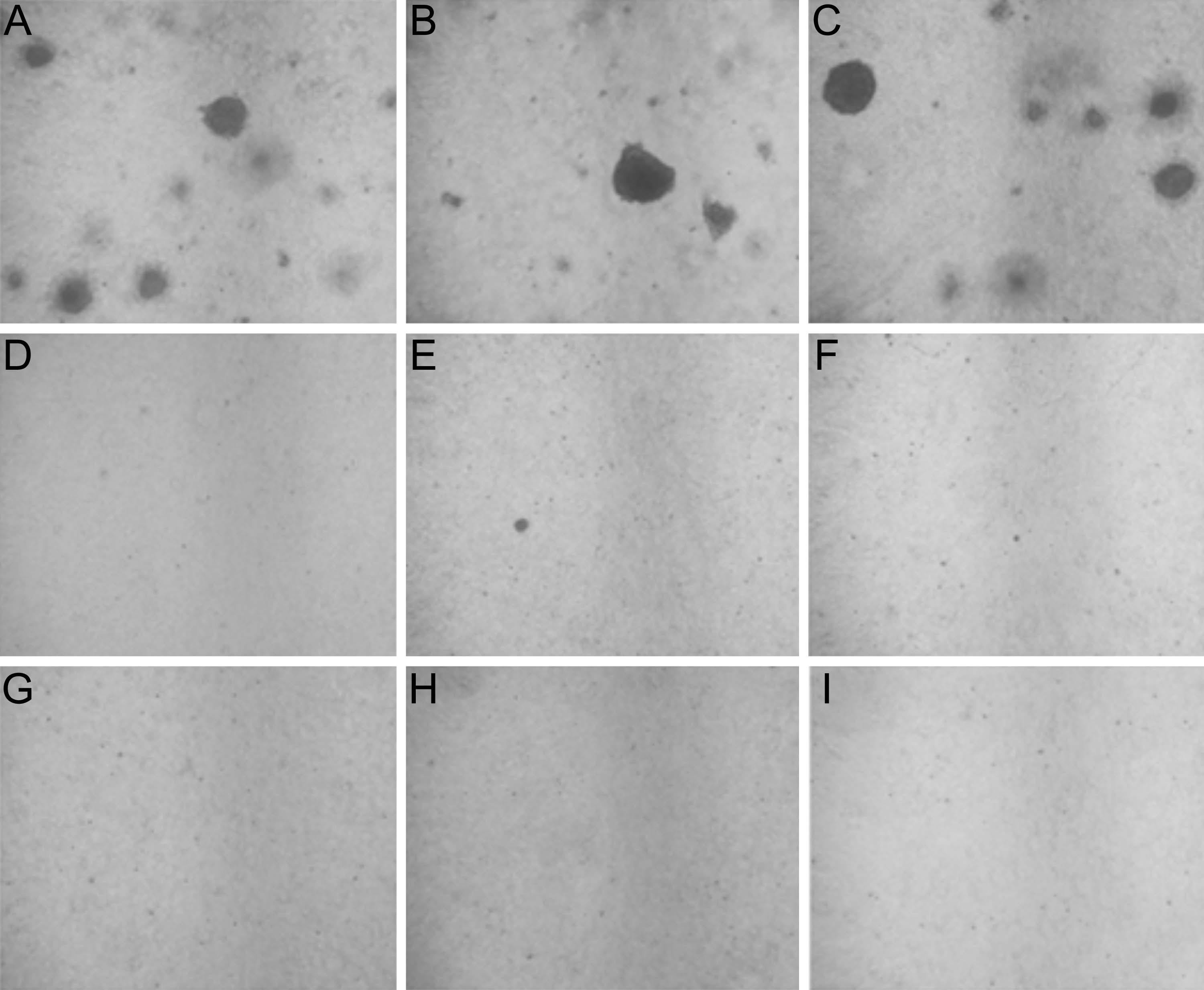

Supplement: Additional file 5 — Figure S5 Overexpression of HOXA1 does not confer the ability to form colonies in soft agar. HaCAT-Control and HaCAT-HOXA1 cells were plated in triplicate wells in 0.4% agar and allowed to grow for 4 weeks. SCC9 cells were used as the positive control. This experiment was reproduced 3 times. Panels A-C represent SCC9 cells, D-F represent HaCAT-Control clones, and G-I correspond to HaCAT-HOXA1 clones. [file 1471-2407-12-146-S5.JPEG]
